# Local release of properdin in the cellular microenvironment: role in pattern recognition and amplification of the alternative pathway of complement

**DOI:** 10.3389/fimmu.2012.00412

**Published:** 2013-01-17

**Authors:** Claudio Cortes, Jennifer A. Ohtola, Gurpanna Saggu, Viviana P. Ferreira

**Affiliations:** ^1^Department of Medical Microbiology and Immunology, College of Medicine and Life Sciences, University of ToledoToledo, OH, USA; ^2^Department Medical Immunology and Microbiology, Medical University of the AmericasWest Indies, Nevis

**Keywords:** properdin, alternative pathway, complement system, pattern recognition, inflammation

## Abstract

Properdin, the only positive regulatory protein of the complement system, acts as both a stabilizer of the alternative pathway (AP) convertases and as a selective pattern recognition molecule of certain microorganisms and host cells (i.e., apoptotic/necrotic cells) by serving as a platform for *de novo* C3b,Bb assembly. Properdin, a highly positively charged protein, normally exists as cyclic dimers (P_2_), trimers (P_3_), and tetramers (P_4_) of head-to-tail associations of monomeric 53 kDa subunits. While most complement proteins are produced mainly in the liver, properdin is synthesized primarily by various cell types, including neutrophils, monocytes, primary T cells, and shear-stressed endothelial cells resulting in properdin serum levels of 4–25 μg/ml. Multiple inflammatory agonists stimulate the release of properdin from stimulated leukocytes into the cellular microenvironment. Concentrated, focused increases in properdin levels may lead to stabilization and initiation of AP convertases, thus greatly amplifying the complement response to a local stimulus. This review highlights current knowledge related to these properties and discusses the implications of properdin production in a pro-inflammatory microenvironment.

## THE COMPLEMENT SYSTEM ALTERNATIVE PATHWAY

The complement system efficiently defends the host from pathogenic microorganisms, facilitates removal of immune complexes, and represents an important link between the innate and adaptive immune systems. Complement comprises three distinct pathways (classical, alternative, and lectin) that converge at the activation of the central protein C3. Complement activation results in direct killing, marking of the target with ligands (C3b, iC3b, C3d) for innate cellular receptors (CR1/CD35, CR2/CD21, CR3, CR4, CRIg, CD46), and numerous adaptive cellular and humoral responses. Sub-products generated during complement activation (C3a and C5a) are important for phagocyte recruitment to infection sites. Although complement is beneficial to the body’s defense system, complement also participates in tissue-damaging processes occurring in many diseases (reviewed in Oksjoki et al., 2007; Holers, 2008).

The alternative pathway (AP) represents a true safeguard system that is initiated in the fluid-phase by spontaneous hydrolysis of the thioester bond in C3 to produce C3(H_2_O), which is functionally and structurally similar to C3b (Pangburn et al., 1981). Binding of factor B to C3(H_2_O) in the presence of factor D generates an unstable fluid-phase C3 convertase (C3(H_2_O),Bb) that digests C3 to generate C3b fragments (Pangburn et al., 1980). C3b fragments possess a labile thioester group that binds covalently to exposed amino or hydroxyl groups on nearby pathogenic as well as host membranes. Once bound to a surface, C3b binds factor B, which is then cleaved by factor D, generating the membrane-bound AP C3 convertase (C3b,Bb) that leads to C3b deposition on the cell surface (Muller-Eberhard and Gotze, 1972; Pangburn, 1998; Rother, 1998). Properdin, the only positive regulatory protein of the complement system, and the focus of this review, extends the half-life of the nascent C3b,Bb convertase (5–10-fold) by forming the complex C3b,Bb,P (Fearon and Austen, 1975; Schreiber et al., 1975), leading to accelerated and efficient amplification of C3b deposition on surfaces (Muller-Eberhard and Gotze, 1972; Pangburn, 1998; Rother, 1998). The AP plays a fundamental role in the amplification of all three pathways because C3b molecules generated by the classical and lectin pathways contribute to generating AP C3 convertases (Harboe and Mollnes, 2008; Gulati et al., 2012). Binding of C3b to the C3b,Bb complex forms the AP C5 convertase (C3b)_2-*n*_,Bb, which is also stabilized by properdin. C5 cleavage forms C5a and C5b (Medicus et al., 1976), initiating formation of the membrane attack complex (C5b-C9) that is common to all complement pathways. Although deposition of C3b occurs on all cells exposed to activated complement, complement regulatory proteins control activation on our own cells to prevent unintended injury (reviewed in Ferreira et al., 2010b; Ricklin et al., 2010).

Unlike the classical and lectin pathways that rely on specific recognition molecules (C1q and mannan binding lectin/ficolins, respectively) for initiation, the AP activates on any surface that lacks the ability to regulate complement. However, the AP does preferentially target selective carbohydrates and amino acids recognized by reactive C3b molecules (reviewed in Pangburn et al., 2008). Moreover, bacterial LPS (Liang-Takasaki et al., 1983; Clardy, 1994), aggregated immunoglobulins (particularly IgA; Hiemstra et al., 1987), and natural antibodies (Ratnoff et al., 1983; Jelezarova and Lutz, 1999; Zhou et al., 2012) can activate the AP independently from the classical or lectin pathways. Recently, properdin has been identified as an initiating pattern recognition molecule of the AP, as will be discussed further.

## PROPERDIN: AN ANCIENT COMPLEMENT REGULATORY MOLECULE OF THE ALTERNATIVE PATHWAY

### PROPERDIN STRUCTURE

Properdin, a highly positively charged protein, exists as cyclic dimers (P_2_), trimers (P_3_), and tetramers (P_4_) of head-to-tail associations of monomeric subunits (Smith et al., 1984; Pangburn, 1989). Each monomer is ~53 kDa (Nolan and Reid, 1990), 26 nm in length × 2.5 nm in diameter (Smith et al., 1984, 1991), contains 442 amino acid residues, and is composed of seven thrombospodin repeat type I (TSR0-TSR6) domains (Higgins et al., 1995; Sun et al., 2004). TSR4 is important in stabilizing the C3b,Bb convertase complex, and TSR5 in C3b and sulfatide binding. TSR3, however, is not required for C3b,Bb stabilization nor sulfatide binding (Higgins et al., 1995). In addition, the properdin monomer contains an N-glycosylation site and 14 C-linked mannosylation sites at tryptophan residues with unknown function (Hartmann and Hofsteenge, 2000). These native forms of properdin bind with a greater affinity to cell-bound C3b,Bb or C3b,B than to cell-bound C3b, but binding to all three molecules is more favorable when bound to a cell membrane versus their respective fluid-phase counterparts (Farries et al., 1988b). Properdin also interacts with C3(H_2_O) (a C3b-like protein; Pangburn and Muller-Eberhard, 1980) and the Ba domain of factor B (Farries et al., 1988a), which is important for AP C3 and C5 convertase formation.

### RE-EMERGING ROLE OF PROPERDIN AS A SELECTIVE RECOGNITION MOLECULE

When properdin was discovered, it was thought to be an initiator of the AP (Pillemer et al., 1954). This controversial view was later replaced by the widely accepted notion that properdin serves as a positive regulator that amplifies the AP by extending the half-life of the C3 and C5 convertases (Fearon and Austen, 1975; Medicus et al., 1976). Recent reports propose that properdin acts as a pattern recognition molecule (as discussed ahead). This view is consistent with the complement initiation function proposed over 50 years ago (Pillemer et al., 1954) and has re-opened the controversy regarding the functions of properdin.

#### Properdin bound to a surface has the potential to initiate complement activation

Hourcade (2006) demonstrated that properdin, covalently bound to a biosensor surface, could subsequently recruit C3b and factor B to form C3b,Bb,P. Importantly, this study also showed that even when properdin binds to surface-bound C3b, properdin can still recruit C3b and factor B to form a new convertase. This goes beyond the “convertase stabilizer” function, in which properdin binds only once the convertase is already formed. Additional evidence supporting the ability of properdin to initiate complement activation (by forming *de novo* C3 convertases on cell surfaces) comes from studies where human embryonic kidney cells (Vuagnat et al., 2000) or *Escherichia coli* (Spitzer et al., 2007) were transfected with a vector expressing a transmembrane form of properdin on the cell surface, turning the cell surface into an activator of the AP.

#### Properdin has been shown to bind to a variety of cell surfaces

Recent studies have reported properdin binding directly to various non-self surfaces: zymosan (Spitzer et al., 2007; Ferreira et al., 2010a), rabbit erythrocytes, *Neisseria gonorrhoeae* (Spitzer et al., 2007), certain *E. coli* strains (Spitzer et al., 2007; Stover et al., 2008), early (Kemper et al., 2008) or late (Xu et al., 2008) apoptotic cells, necrotic cells (Xu et al., 2008; Ferreira et al., 2010a), live human leukemia T cell lines (Kemper et al., 2008), normal human proximal tubular epithelial cells (Gaarkeuken et al., 2008), Chinese hamster ovary cells (Kemper et al., 2008), neutrophils (Wirthmueller et al., 1997; Camous et al., 2011), and cartilage oligomeric matrix protein (Happonen et al., 2010). Furthermore, bound properdin serves as a platform for *de novo* C3b,Bb assembly, leading to C3 cleavage and complement activation on these surfaces, suggesting that properdin may serve as a pattern recognition molecule for AP initiation on targets.

#### To study the specificity of properdin-target interactions the physiological forms of properdin (*P_2_–P_4_*) should be separated from aggregated (“activated”) properdin

Biochemical studies of serum-derived pure properdin indicate that non-physiological high molecular weight, highly positively charged polymers (known as P_*n*_ or “activated” properdin) form during long term storage and freezing/thawing (Farries et al., 1987; Pangburn, 1989). Although “activated” properdin (or P_*n*_) retains the ability to stabilize AP convertases, it possesses the abnormal capacity to activate complement in solution (consumption of complement; Pangburn, 1989) and bind non-specifically to surfaces such as live T cells (Ferreira et al., 2010a) and *Nesseriae* (Agarwal et al., 2010). The studies mentioned in the previous section (except Agarwal et al., 2010; Ferreira et al., 2010a; Cortes et al., 2011) and parts of other studies (Kemper et al., 2008; Xu et al., 2008; Camous et al., 2011) were carried out with unfractionated pure properdin potentially containing aggregates. Studies using physiological forms of properdin (P_2_–P_4_) separated from non-physiological aggregates, by ion exchange and/or size exclusion chromatography, found native properdin does not bind to some previously described surfaces, such as rabbit erythrocytes, live Jurkat cells (Ferreira et al., 2010a) and *Neisseria* sp. (Agarwal et al., 2010). However, native properdin forms do bind to necrotic cells, yeast cell wall components (Ferreira et al., 2010a), *Chlamydia pneumoniae *(Cortes et al., 2011), and activated platelets (Saggu et al., 2012), suggesting it is a highly selective recognition molecule. In addition, neutrophil-derived native/physiological properdin can bind to apoptotic T cells (Kemper et al., 2008) and neutrophils (Wirthmueller et al., 1997; Camous et al., 2011), while properdin, in the context of C3-deficient serum can bind to dying cells (Xu et al., 2008). Interestingly, T cell-derived properdin is ~100 times more active than serum properdin (Schwaeble et al., 1993) when tested in a traditional AP hemolytic assay, but the molecular mechanisms involved in the increased activity remain unknown. Although it has been speculated that serum-derived, unfractionated properdin (containing aggregated “activated” properdin) may be similar to native neutrophil- or T cell-derived properdin, biochemical experimental evidence is lacking. Moreover, activated properdin forms (P_*n*_), are not normally in circulation (or are tightly controlled) since their presence leads to systemic complement activation and consumption (Pangburn, 1989). Based upon available experimental evidence, future studies should be carried out only with native properdin forms (separated from “activated” properdin), or with fresh leukocyte-derived properdin, in order to effectively determine specific interactions between properdin and surfaces and not over-estimate the role of properdin (due to aggregates) in complement activation.

Surface-bound properdin may lead to complement activation by recruiting C3b molecules derived from different sources. For instance, it is possible that properdin binds C3b generated during the activation of any of the three complement pathways or recruits soluble C3(H_2_O) (a C3b-like molecule) to form a membrane-bound C3(H_2_O),Bb convertase. We have recently determined that physiological forms of properdin bound to activated platelets, but not resting platelets, recruits both C3(H_2_O) and factor B, generating a functional C3(H_2_O),Bb convertase that promotes complement activation on platelets (Saggu et al., 2012), whereas platelet-bound C3(H_2_O) alone has been shown to not produce a functional convertase (Hamad et al., 2010).

## THE ROLE OF PROPERDIN IN THE LOCAL MICROENVIRONMENT

### SOURCES OF PROPERDIN

**FIGURE 1:**
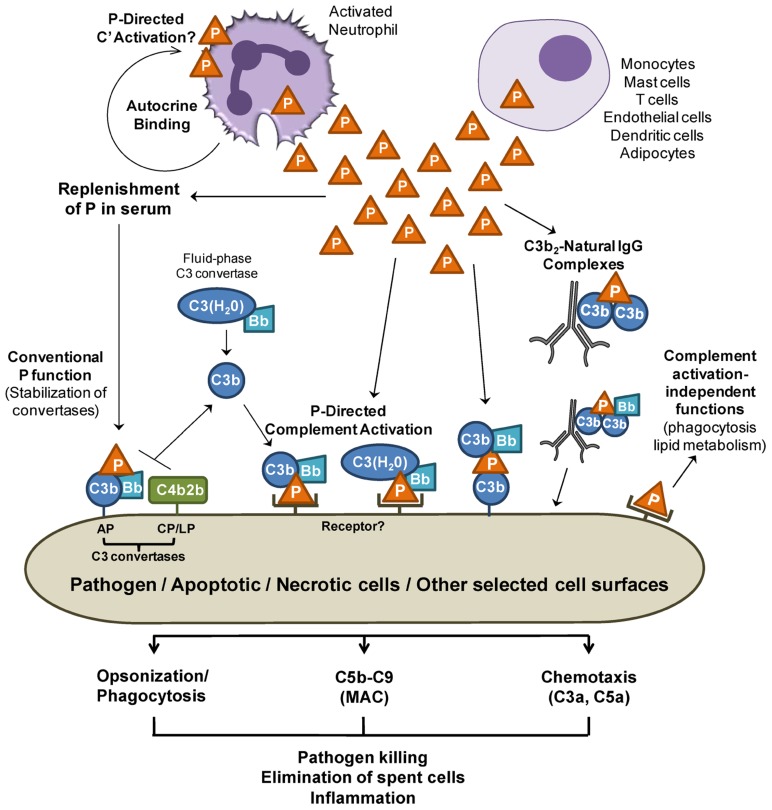
**Release of properdin in the local microenvironment**. Properdin (P) released by immune cells may directly bind to surfaces (pathogens and cells) and promote AP complement activation. Properdin may recruit C3b or C3(H_2_O) to form a C3 convertase and further promote C3b deposition on surfaces (P-mediated complement activation). Sources of C3b may be derived from C3 convertases of the alternative pathway (AP), lectin (LP), or classical pathways (CP). C3(H_2_O) derived from “tick over” C3 hydrolysis may also bind to cell-bound properdin, forming a C3(H_2_O),Bb convertase on the cell. In addition, properdin can bind to C3b on surfaces and recruit additional C3b and factor B, generating new convertases. Properdin that does not encounter a nearby cell surface may lose the ability to bind to surfaces directly soon after it is in contact with blood, therefore preventing unwanted properdin-mediated complement damage in surrounding areas while keeping the conventional function of stabilizing the C3 and C5 convertases of the AP. Properdin-mediated complement activation may participate in opsonization, MAC deposition, and C3a and C5a release, which are important processes in inflammatory immune responses. Finally, locally released properdin may carry out functions that are independent from complement activation/amplification. For simplicity, the orange triangle represents a properdin trimer.

Unlike most other complement proteins, which are produced mainly in the liver, properdin is synthesized by various cell types (**Table 1**, **Figure 1**) resulting in properdin serum levels of 4–25 μg/ml (Pangburn, 1989; Nolan and Reid, 1993; Fijen et al., 1999; Schwaeble and Reid, 1999; Xu et al., 2008). Multiple inflammatory agonists, such as TNF-α, C5a, or fMLP, stimulate the release of properdin (**Table 1**) into the pro-inflammatory microenvironment to induce local AP activation.

**Table 1:** List of sources of properdin.

Cellular Source		Form	Stimulus	Reference
**Primary cells**				
Monocytes		protein	constitutive	Whaley (1980)
Dendritic cells				
	*Monocyte-derived*	mRNA, protein	constitutive	Reis et al. (2006), Li et al. (2011)
	*Dermal*	mRNA	constitutive	Li et al. (2011)
	*Langerhans*	mRNA	constitutive	Li et al. (2011)
	*Myeloid*	mRNA	constitutive	Li et al. (2011)
	*Plasmacytoid*	mRNA	constitutive	Li et al. (2011)
Primary T cells		mRNA	constitutive	Schwaeble et al. (1993)
Mast cells		protein	constitutive	Stover et al. (2008)
Granulocytes		protein mRNA, protein	TNF-α, TNF/fMLP, PMA TNF-α, C5a, IL-8, fMLP	Wirthmueller et al. (1997), Camous et al. (2011) Wirthmueller et al. (1997)
Endothelial cells		mRNA, protein	laminar shear stress	Bongrazio et al. (2003)
Adipocytes		mRNA, protein	constitutive	Peake et al. (1997), Pattrick et al. (2009)
**Cell lines**				
H-9 (T cell)		mRNA	constitutive	Schwaeble et al. (1993)
HuT78 (T cell)		mRNA	constitutive	Schwaeble et al. (1993)
Jurkat (T cell)		mRNA	constitutive	Schwaeble et al. (1993)
T-ALL (T cell)		mRNA	constitutive	Schwaeble et al. (1993)
HL-60 (promyelocyte)		protein	DMSO	Farries and Atkinson (1989)
U-937 (monocyte)		Protein	PMA, LPS, IFN-γ	Minta (1988)
Monocyte Mono Mac6		mRNA, protein	IFN-γ (mRNA only), IL-1β, LPS, TNF-α, PMA	Schwaeble et al. (1994)
3T3-L1 adipocytes		mRNA	constitutive	Peake et al. (1997)

### LOCAL RELEASE OF PROPERDIN

Concentrated transient increases in local properdin levels due to cell production (i.e., T cells, monocytes, and neutrophils) would likely lead to stabilization (reviewed in Schwaeble and Reid, 1999) and initiation of the AP convertases, thus greatly amplifying the complement response to a local stimulus, in particular because these cells also synthesize the other complement proteins necessary for activation.

The complement system plays a role in the clearance of dead/dying cells through opsonization and promotion of phagocytosis (Flierman and Daha, 2007; Trouw et al., 2008). Properdin released by phagocytes binds to apoptotic and necrotic cells (Kemper et al., 2008; Xu et al., 2008), and this may aid in their removal directly or through properdin-mediated complement activation (**Figure 1**). Likewise, local release of properdin may opsonize and kill microorganisms by directly promoting their phagocytosis or through properdin-mediated complement opsonization and killing. Native properdin forms bind directly to *C. pneumoniae* and promote AP-mediated complement activation, with possible consequences in infectivity (Cortes et al., 2011) and in chronic inflammation found in atherosclerosis. Properdin-mediated complement activation may also be important for further recruitment of pro-inflammatory cells to infection sites. Concomitantly, properdin may play other direct roles, independent from complement activation, influencing lipid metabolism (Gauvreau et al., 2012).

At sites of inflammation where many different properdin-producing cells are in close proximity and cytokine release and complement activation occurs, neutrophils rapidly secrete properdin upon degranulation stimuli (**Table 1**). Endogenous native properdin has been detected on the surface of isolated, non-stimulated neutrophils (Wirthmueller et al., 1997) and TNF/fMLP-stimulated neutrophils (Camous et al., 2011), independently from C3 (Camous et al., 2011). Unfractionated properdin (known to contain non-physiological complement-activating aggregates, as described above), when incubated with isolated resting neutrophils, promotes complement activation on neutrophil membranes (Camous et al., 2011), and when added to whole blood, induces the formation of platelet–leukocyte aggregates (Ruef et al., 2008). Interestingly, pro-inflammatory and coagulation-induced stimuli allow neutrophils to activate the AP in an autocrine or paracrine fashion, despite the presence of membrane-bound complement regulatory proteins, on neutrophil surfaces (Camous et al., 2011). The exact mechanism of complement activation on neutrophils remains to be determined and properdin-mediated initiation is possible. Complement activation on neutrophils results in increased release of complement products such as C5a fragments and the MAC complex which could further activate neutrophils, endothelial cells, or other cells in close contact with adherent neutrophils further contributing to a pro-inflammatory microenvironment.

*In vivo* studies using properdin-deficient mice have revealed important roles for properdin in disease models including septic and non-septic shock (Ivanovska et al., 2008; Stover et al., 2008), various arthritis models (Dimitrova et al., 2010, 2012; Kimura et al., 2010), and abdominal aortic aneurism (AAA; Zhou et al., 2012). Because properdin-deficient mice are protected from the severity of certain diseases (Ivanovska et al., 2008; Kimura et al., 2010; Zhou et al., 2012), efforts to understand the consequences of therapeutically inhibiting properdin (Gupta-Bansal et al., 2000; Kimura et al., 2010) and the contribution of locally synthesized properdin in the disease pathogenesis are being evaluated.

### POTENTIAL BINDING LIGANDS FOR PROPERDIN ON SURFACES

Properdin is a highly positively charged protein (isoelectric point >9.5). Properdin may interact directly with surfaces by recognizing glycosaminoglycan (GAG) chains of surface proteoglycans on proximal tubular epithelial cells (Zaferani et al., 2011) and T cells (Kemper et al., 2008). Candidate sulfated GAGs shown to interact with properdin include heparin (Yu et al., 2005), heparan sulfate (Kemper et al., 2008; Zaferani et al., 2011), dextran sulfate (Holt et al., 1990), fucoidan (Holt et al., 1990), and chondroitin sulfate (Kemper et al., 2008). Interestingly, Holt et al. (1990) demonstrated differences between the sulfated glycoconjugate binding properties of native and activated (P_*n*_) properdin. While both native and activated properdin bind to dextran sulfate (M_r_ 500,000) and fucoidan, only the “activated” form of properdin binds to chondroitin sulfate C, heparin, and dextran sulfate (M_r_ 5,000). Other ligands for properdin on cells include DNA on late apoptotic and necrotic cells (Xu et al., 2008), and bacterial LPS and lipooligosaccharide (Kimura et al., 2008). All cell surface molecules (identified to date), shown to interact directly with properdin on cells, are negatively charged (with the exception of the convertase proteins of the AP). Additional studies are needed for identifying the receptors for properdin on other surfaces on which properdin has been found to bind, as discussed previously herein.

### BINDING OF PROPERDIN TO SURFACES IS REGULATED IN SERUM

Binding of purified physiological forms of properdin to certain surfaces (zymosan, necrotic cells, and *C. pneumoniae*) is inhibited by normal human serum in a dose-dependent manner (Ferreira et al., 2010a; Cortes et al., 2011). In agreement with these results, binding of unfractionated pure properdin to apoptotic T cells was also inhibited in the presence of serum (Kemper et al., 2008), and direct binding of properdin to zymosan and *E. coli* was not detected in the context of normal human serum or lepirudin-anticoagulated plasma unless C3 components bound first (Harboe et al., 2012). Therefore, yet-to-be identified inhibitors of this interaction may exist in serum. Local production of properdin transiently elevates the concentration in close proximity to the cells producing it, while properdin that leaves the microenvironment of production will be progressively inhibited as a regulatory mechanism. Tight regulation of the ability of properdin to bind to surfaces would be expected in order to prevent unwanted properdin-mediated complement activation and damage at more distant/bystander cell surfaces. As mentioned, properdin binds DNA and sulfated glycoconjugates. Thus, fluid-phase forms of DNA (Rhodes et al., 2006) or glycoproteins could potentially serve as regulators of properdin/surface interactions once properdin has left the microenvironment of the cells producing it (i.e., neutrophils). Studies aimed at identifying putative serum-derived inhibitors of the interaction between properdin and cell surfaces are necessary.

## FINAL REMARKS

The complement system is an essential component of the innate immune system that participates in elimination of pathogens and altered host cells. The important role of the local production of complement components and their role in the inflammatory microenvironment is an important emerging field. Properdin, the only known positive regulatory protein of the complement system, is produced by various cell types. This review describes the role of properdin as a stabilizer of AP convertases and as a selective pattern recognition molecule, highlighting its function as an activator of the AP on surfaces to which it binds (pathogens, host cells). Future studies aimed at identifying the receptor(s) that bind properdin on pathogens and host cells, the factors that contribute to regulation of properdin binding to surfaces in serum, the differences between serum-derived and leukocyte-derived properdin, and the role of properdin in the pro-inflammatory microenvironment, specifically in exacerbating or controlling inflammatory diseases, will significantly contribute to determining under which scenarios the therapeutic inhibition of specific properdin functions may be warranted.

## Conflict of Interest Statement

The authors declare that the research was conducted in the absence of any commercial or financial relationships that could be construed as a potential conflict of interest.
